# RNA splicing and cardiovascular disease: a guide for cardiologists

**DOI:** 10.1093/eurheartj/ehag374

**Published:** 2026-06-05

**Authors:** Dakota R Hunt, Shambhabi Chatterjee, Leslie A Leinwand, Thomas Thum

**Affiliations:** BioFrontiers Institute, University of Colorado Boulder, 3415 Colorado Avenue, Boulder, CO 80303, USA; Department of Biochemistry, University of Colorado Boulder, Boulder, CO 80303, USA; Institute of Molecular and Translational Therapeutic Strategies (IMTTS), Hannover Medical School, Carl-Neuberg-Straße 1, Hannover 30625, Germany; BioFrontiers Institute, University of Colorado Boulder, 3415 Colorado Avenue, Boulder, CO 80303, USA; Department of Molecular, Cellular, and Developmental Biology, University of Colorado Boulder, Boulder, CO 80309, USA; Institute of Molecular and Translational Therapeutic Strategies (IMTTS), Hannover Medical School, Carl-Neuberg-Straße 1, Hannover 30625, Germany

**Keywords:** Alternative splicing, Cardiovascular disease, Cardiac development, RNA-binding proteins, RNA isoforms, Non-coding RNA

## Abstract

Alternative splicing (AS) is a fundamental RNA processing mechanism, which generates different RNA transcripts and consequently different protein isoforms from a single gene. This increases the diversity of proteins within an organism and can fine-tune biological processes. This review examines how cardiac-enriched RNA-binding proteins establish heart-specific splicing programs governing aspects of cardiac development, function, and disease. Developmentally, coordinated sarcomeric isoform switches underpin the foetal-to-adult transition and further isoform rewiring in ion channel and kinase genes determine electrophysiology and excitation–contraction coupling. AS contributes to the pathogenesis of several cardiomyopathies and emerging datasets suggest that pathological hypertrophy engages distinct splicing signatures compared with physiological hypertrophy. This review summarizes diagnostic and prognostic opportunities arising from bulk, long-read, and single-cell/nucleus transcriptomics, which resolve cell type-specific isoforms and disease-associated switches. Circulating RNA biomarkers (including splice ratios and circularRNAs) may signify myocardial remodelling and arrhythmic risk. Integrative approaches that link AS with proteomics and genomics improve variant interpretation, reveal previously unannotated protein isoforms, and enable tracking of disease progression and therapy response. Finally, an outline of therapeutic strategies to modulate AS in cardiovascular disease (CVD), including antisense oligonucleotides, small molecules, and genome-editing modalities (CRISPR, base, and prime editing), is provided. The major challenges that remain before splice-targeting therapeutics can be targeted to treat cardiovascular disease are highlighted. Lessons from neuromuscular indications establish clinical feasibility of splicing correction and motivate translation to cardiology. Together, mechanistic insight, biomarker development, and therapeutic innovation position RNA splicing as a tractable axis for precision cardiovascular medicine.

## Graphical Abstract

**Graphical Abstract:**
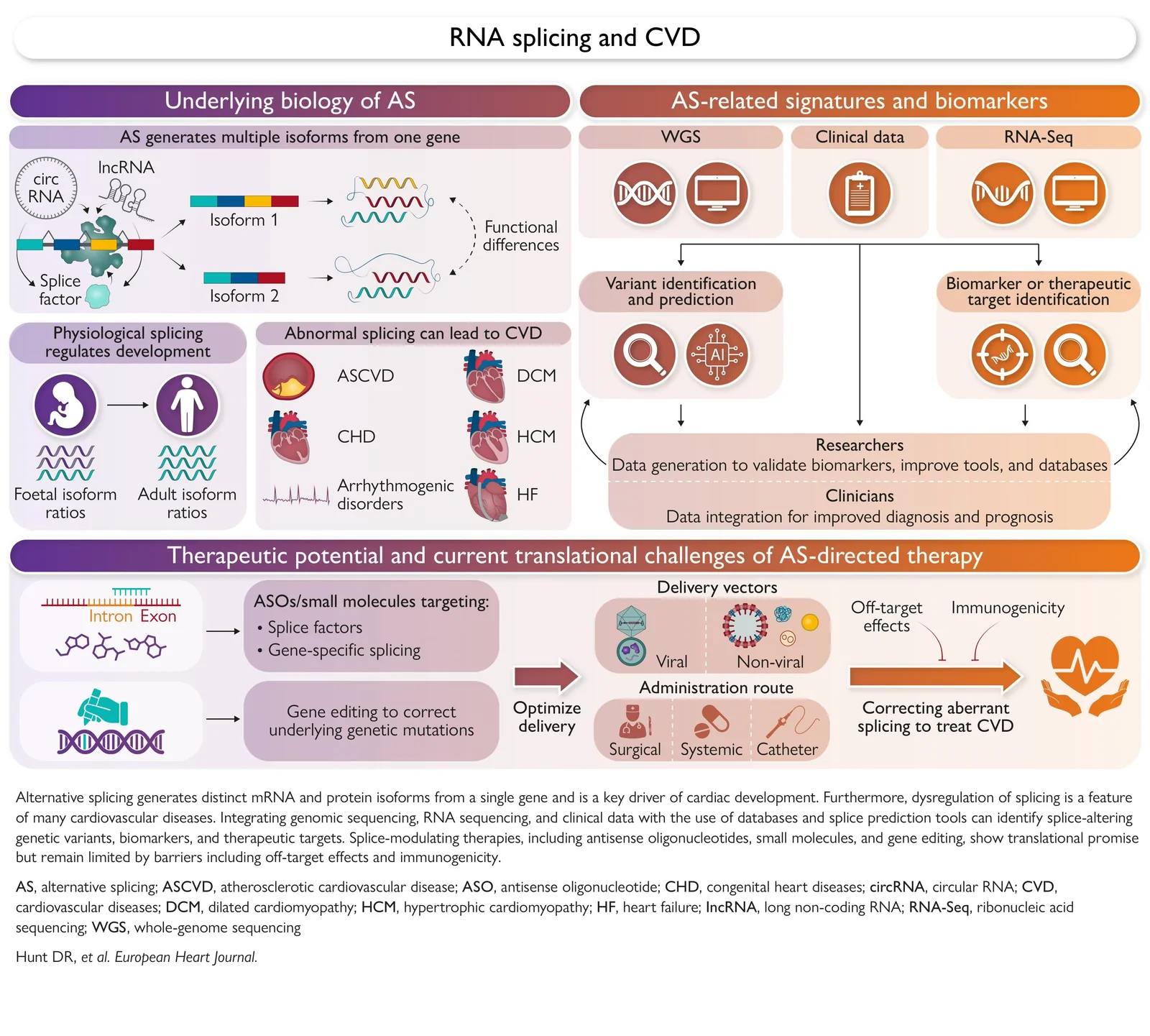
Created in BioRender. Chatterjee, D. S. (2026) https://BioRender.com/9bwrwdr.

## Introduction

The flow of genetic information from DNA to messenger RNA (mRNA) and ultimately to a functional protein is a complex process that is regulated at multiple steps.^1^ Alternative splicing (AS) is a fundamental mechanism that allows a single gene to produce multiple mRNA and protein variants by including or skipping different protein-coding exons during precursor mRNA (pre-mRNA) processing.^2^ Pre-mRNA splicing is orchestrated by the spliceosome, a massive RNA–protein complex that facilitates intron removal and joining of exons through highly conserved GU–AG splice sites. AS can be regulated at different stages of spliceosome assembly and by different mechanisms, including cis-acting RNA elements embedded in pre-mRNA and trans-acting regulatory proteins. Strikingly, this AS process also orchestrates the formation of various non-coding transcripts such as microRNAs (miRNAs^3^) and long non-coding RNAs (lncRNAs),^4^ which do not translate to a functional protein but nevertheless can act as master regulators of gene expression.^5^ Together, these components determine which exons are included or skipped in the final mRNA.

AS greatly expands proteome diversity—over 90% of human genes generate more than one isoform—and enables specialized protein functions in different tissues and conditions. In the multicellular heart, distinct splicing patterns help define the various cardiac lineages and the differences between foetal and adult cardiac cells.^6^ Structural and regulatory genes undergo developmental splice switches, ensuring the adult heart expresses isoforms optimized for excitation–contraction coupling (E-C coupling)^7^ and myocardial mechanics, while foetal isoforms are silenced.^8–10^ Genetic mutations in cis- or trans-acting regulatory elements can lead to abnormal splicing patterns and the development of disease. Similarly, environmental stressors may lead to dysregulated expression of regulatory factors and widespread downstream splicing changes. Aberrant^11,12^ or foetal splice variants^13^ often reappear in pathologic hypertrophy and heart failure (HF), suggesting regression to developmental programs.^12,14^ Changes in mRNA splicing have been documented across virtually every stage of cardiac pathology.^15^ Notably, certain pathological splice signatures are closely entwined with cardiac phenotype and could be leveraged clinically for early diagnosis or risk stratification.

This review begins by summarizing the basics of RNA splicing, where standard concepts and terminologies are summarized in Glossary Box 1. Subsequently, how AS events play crucial roles in heart development and disease progression and their application in the clinic as biomarkers is highlighted. The development of tools available for the identification of AS events and prediction of splice-altering variants are outlined. Finally, the work on targeted therapies pertaining to AS and the barriers to their clinical translation are discussed. To help readers navigate technical terminology, a list of abbreviations along with their definitions is provided (*Table 1*).

Glossary Box **AS—Alternative splicing:** A post-transcriptional process in which different combinations of exons are joined to produce multiple mRNA isoforms from a single gene.

**ASO—Antisense oligonucleotide:** A short synthetic nucleic acid designed to bind a specific RNA sequence and alter its processing, stability, or splicing.

**Back-splice junction:** The unique sequence junction created by back-splicing that distinguishes a circular RNA from its linear RNA counterpart.

**Cis-acting elements:** sequence motifs within pre-mRNA that guide splice site selection or binding of sequence-specific RNA-binding proteins e.g. 5′ splice site (donor site); 3′ splice site (acceptor site).

**circRNA—circular RNA:** A covalently closed RNA molecule generated by a non-canonical splicing event in which the RNA ends are joined together to create a unique back-splice site. The circular transcript is highly stable as it is not degraded as fast as the linear transcripts.

**CRISPR:** A programmable gene-editing system that can be used to modify genomic sequences, including correcting mutations or splice-regulatory regions that cause aberrant splicing.

**Exon:** Segment of a gene that code for a protein or a functional RNA (non-coding RNA transcript).

**Intron:** Introns are the regions of a gene that are spliced out before the mRNA is translated into a protein. They are found between exons.

**Isoform:** A distinct mRNA or protein variant of a gene that is produced through alternative splicing or other regulatory mechanisms.

**lncRNA—Long non-coding RNA:** A non-coding RNA transcript longer than 200 nucleotides that does not encode a protein but can regulate gene expression, including RNA splicing.

**miRNA—microRNA:** A small non-coding RNA that typically represses gene expression by binding target mRNAs and reducing their translation or stability.

**Motif:** A short, recurring sequence pattern in DNA, RNA, or protein that has a specific biological function—such as binding site of a protein, defining a splice site, or regulating transcription.

**ncRNA—non-coding RNA:** RNA transcripts that are transcribed from DNA but do not encode proteins and act as master regulators of cellular function. Major classes include microRNAs, long non-coding RNAs, and circular RNAs.

**pre-mRNA—Precursor mRNA:** The initial RNA transcript made from DNA that still contains both exons and introns and must be spliced before becoming a mature mRNA.

**RBP—RNA-binding protein:** Proteins that bind RNA and regulate splicing, stability, localization, and translation.

**snRNP—Small nuclear ribonucleoprotein:** A core RNA–protein subunit of the spliceosome that recognizes splice sites and helps catalyse intron removal.

**Splice site:** A specific nucleotide sequence at the exon–intron boundary recognized by the spliceosome. The two main types are the 5′ splice site (donor) and the 3′ splice site (acceptor), which guide accurate intron removal and exon joining.

**Spliceosome:** A large ribonucleoprotein complex (U1, U2, U4/U6, U5 snRNPs) that catalyses intron removal and exon ligation.

**Trans-acting regulatory factors:** These are RNA-binding proteins that recognize cis-elements and direct splice-site choice. Some examples include SR proteins (e.g. SRSF1–SRSF12) that typically promote exon inclusion and hnRNP proteins (e.g. hnRNPA1, hnRNPC, and hnRNPK), which often promote exon skipping.

**Transcriptional regulators:** Proteins or RNA molecules that control the rate, timing, and specificity of gene transcription. They act by binding to DNA, chromatin, or components of the transcriptional machinery to either activate or repress gene expression. Major classes include transcription factors, co-activators, co-repressors, chromatin remodelers, and epigenetic modifiers.

**Table 1:** List of abbreviations

AAV	Adeno-associated virus
ACTN4	Alpha-actinin-4
AI	Artificial intelligence
AS	Alternative splicing
ASOs	Antisense oligonucleotides
CACNA1C	Alpha-1 subunit of a voltage-dependent calcium channel
CaMKIIδ	Calcium/calmodulin-dependent kinase type II subunit-δ
CASQ2	Calsequestrin 2
CELF	CUGBP and Elav-like family
CHD	Congenital heart disease
circANRIL	Circular antisense non-coding RNA in the INK4 locus
circFhod3	Circular formin homology domain containing 3
circLONP2	Circular RNA derived from the LONP2 gene
circNPHP4	Circular RNA derived from the NPHP4 gene
circRNA	Circular RNA
circSLC8A1	Circular solute carrier family 8 member A1
circStrn3	Circular Striatin-3
circTtn/cTTN1	Circular Titin
CRISPR	Clustered regularly interspaced short palindromic repeats
CUGBP1/2	CUG-binding protein ½
CVD	Cardiovascular disease
DCM	Dilated cardiomyopathy
DMD	Duchenne muscular dystrophy
DSP	Desmoplakin
EVs	Extracellular vesicles
FDA	U.S. Food and Drug Administration
FLNC	Filamin C
FOXP3Δ2	Forkhead box P3 delta 2
FNDC3b	Fibronectin type III domain containing 3B
GATA4	GATA-binding protein 4
HF	Heart failure
HFpEF	Heart failure with preserved ejection fraction
HCM	Hypertrophic cardiomyopathy
hiPSC-CMs	Human induced pluripotent stem cell-derived cardiomyocytes
HIPK3	Homeodomain-interacting protein kinase 3
hnRNP F/A0	Heterogeneous nuclear ribonucleoprotein F (hnRNP F)
ICM	Ischaemic cardiomyopathy
IRI	Ischaemia reperfusion injury
KATMAP	Knockdown activity and target models from additive regression predictions
KCNH2	Potassium voltage-gated channel subfamily H member 2
KCNQ1	Potassium voltage-gated channel subfamily Q member 1
LDL	Low density lipoprotein
LMNA	Lamin A/C
lncRNA	Long non-coding RNA
LONP2	Lon peptidase 2, peroxisomal
LVAD	Left ventricular assist device
MBNL1	Muscleblind-like 1
MEF2A	Myocyte enhancer factor 2A
MEF2D	Myocyte enhancer factor 2D
MI	Myocardial infarction
MICRA	Myocardial infarction-associated circular RNA
miRNA	MicroRNA
MR	Mendelian randomization
mRNA	Messenger RNA
MYBPC3	Myosin-binding protein C3
MYL6	Myosin light chain 6
MYH7	Myosin heavy chain 7
ncRNA	Non-coding RNA
NCX1	Sodium/calcium exchanger protein 1
NPHP4	Nephro-cystin-4 gene
ONT	Oxford nanopore technology
PacBio	Pacific biosciences
PCBP1	Poly(rC)-binding protein 1
PKM	Pyruvate kinase M
pre-mRNA	Precursor mRNA
QKI	Quaking
QTL	Quantitative trait loci
RBFOX1	RNA-binding fox-1 homolog 1
RBFOX2	RNA-binding fox-1 homolog 2
RBM5	RNA-binding motif protein 5
RBM10	RNA-binding motif protein 10
RBM20	RNA-binding motif protein 20
RBM24	RNA-binding motif protein 24
RBPs	RNA-binding proteins
RNP	Ribonucleoprotein
RNA-Seq	RNA-sequencing
RS-domain	Arginine and serine-domain
RYR2	Ryanodine receptor 2
SCN5A	Sodium voltage-gated channel alpha subunit 5
scRNA-Seq	Single-cell RNA-sequencing
SF	Splicing factor
SMA	Spinal muscular atrophy
SMART-Seq	Switching mechanism at the 5′ end of the RNA template sequencing
SMN2	Survival motor neuron 2
snRNPs	Small nuclear ribonucleoproteins
SPLiT-Seq	Split-pool ligation-based transcriptome sequencing
SRSF1/10	Serine and arginine-rich splicing factor 1/10
SS	Splice site
STAT3β	Signal transducer and activator of transcription 3 beta
TARP	Talipes equinovarus, atrial septal defect, robin sequence and persistence of the left superior vena cava
TBX5	T-box transcription factor 5
TCF3	Transcription factor 3
TNNI3	Troponin I3
TNNT2	Troponin T2, cardiac type
TPM1	Tropomyosin 1
TPM3	Tropomyosin 3
TRDN/Trdn	Triadin
TRDN-AS	Triadin antisense
TTN	Titin
TTNtv	Titin truncation variants
TWAS	Transcriptome-wide association studies
VLPs	Virus-like particles
VSCMs	Vascular smooth muscle cells
ZIC3	Zinc finger protein of the cerebellum 3
ZRANB2	Zinc finger RANBP2-type containing 2

## Molecular basis of RNA splicing in the heart

### Constitutive vs alternative splicing

**Figure 1:**
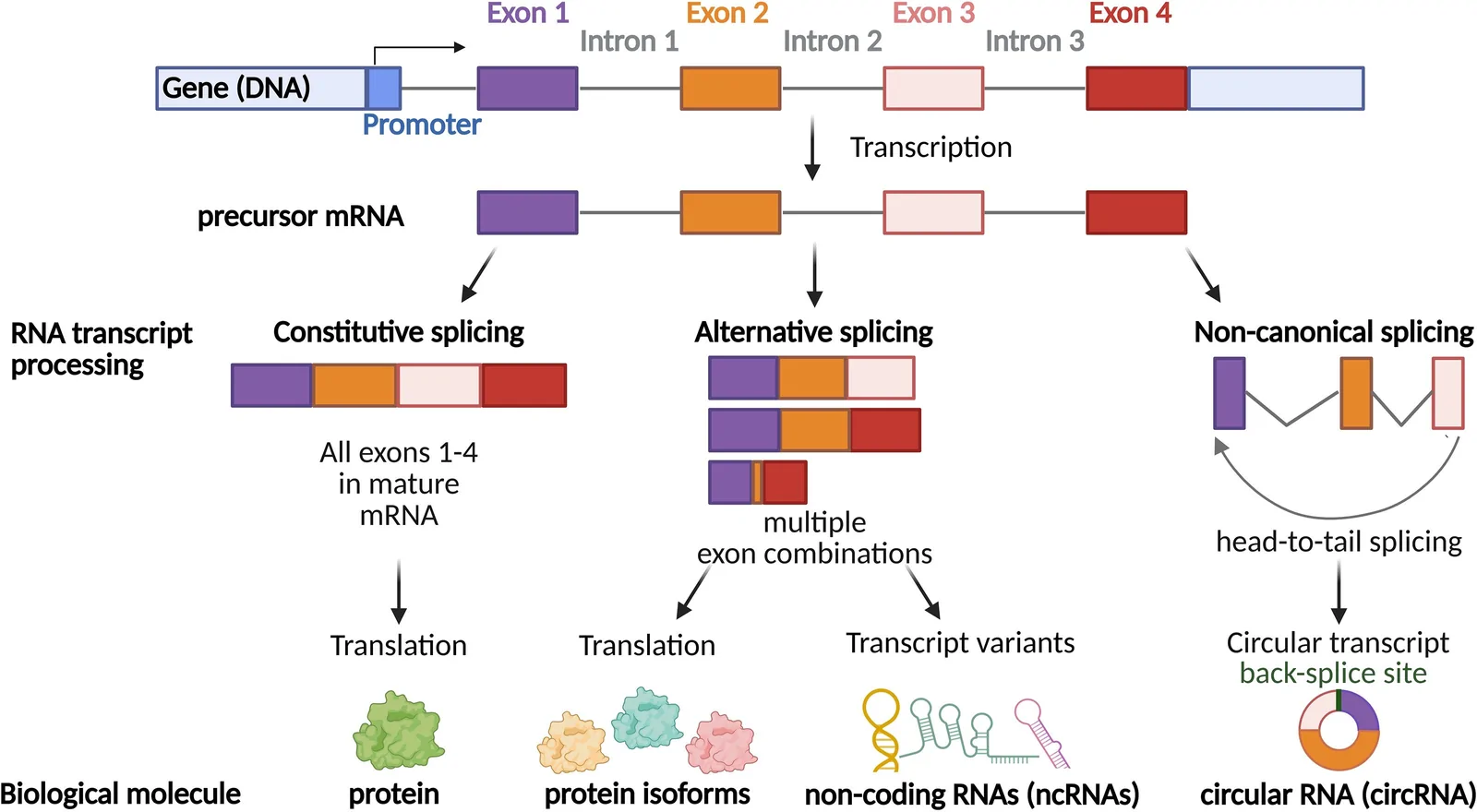
Overview of RNA processing in the cell. The flow of genetic information from DNA to precursor RNA consisting of introns and exons to messenger RNA (only exons) via constitutive or alternative splicing events are depicted. The mRNA is finally translated to a functional protein (single or multiple isoforms). Alternative splicing events can also give rise to long non-coding RNA (lncRNA) transcripts (more details in *Figure 2*). The non-canonical back splicing event leading to formation of circular RNA (circRNA) with a unique back-splice site, which is another class of non-coding RNA with no/limited translation potential. Created in BioRender. Chatterjee, D. S. (2026) https://BioRender.com/8q82mh6

**Figure 2:**
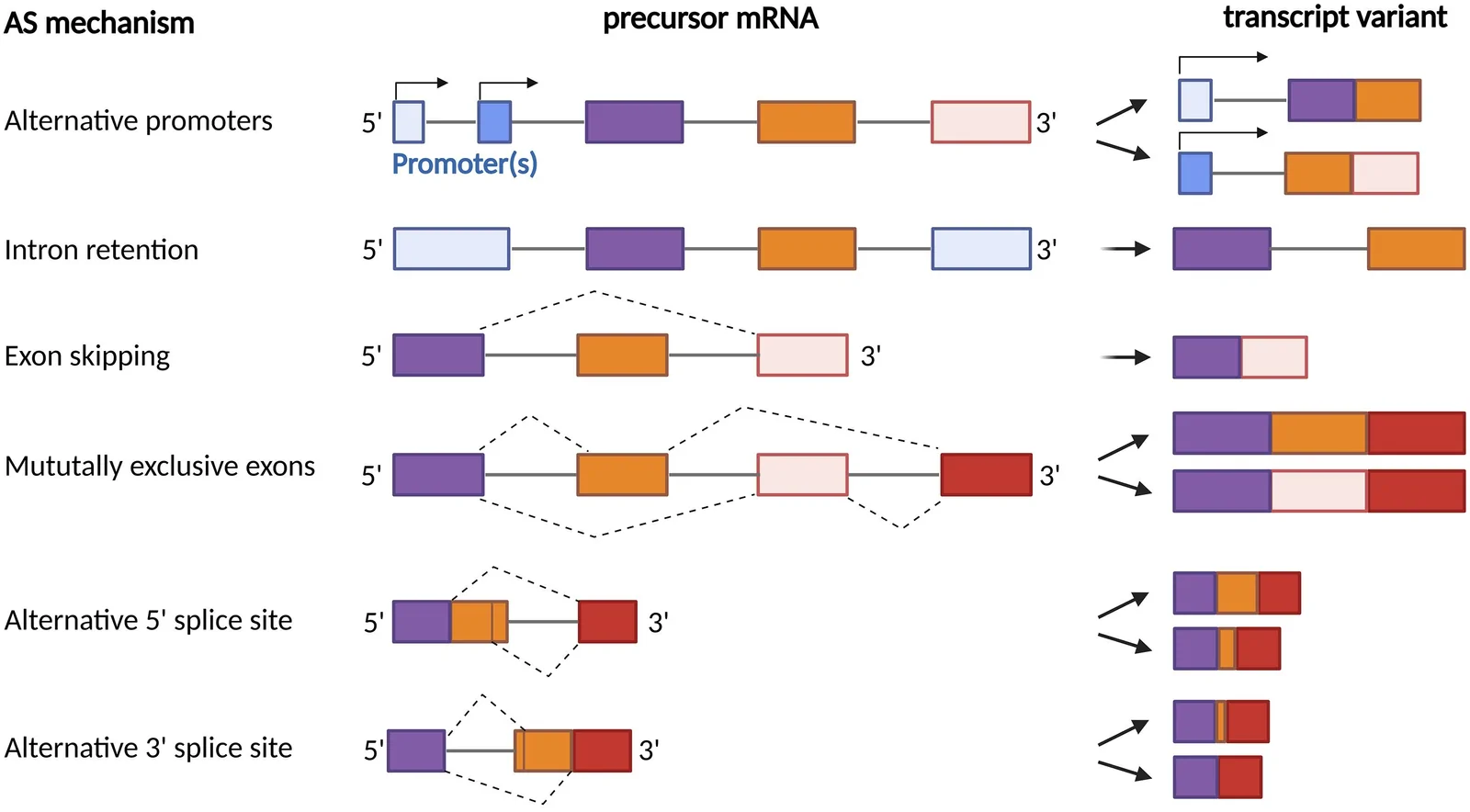
Mechanisms of Alternative splicing. Several mechanisms of alternative splicing can occur within the cell giving rise to various mRNA molecules from the same DNA sequence. Alternative promoter: transcription is initiated via different promoter regions. Intron retention: an intron is included in the mRNA. Exon skipping: most frequent type of alternative splicing in mammalian cells, where an exon is completely skipped. Mutually exclusive exons: only one of two exons is removed, but not both. Alternative donor (5′) or acceptor (3′) site: a different 5′ or 3′ splice junction site is used for alternative splicing which changes the boundary of the exons. Each of these lead to a different protein isoform upon translation. Created in BioRender. Chatterjee, D. S. (2026) https://BioRender.com/ztfh3vi

Splicing is regulated by the spliceosome, a complex molecular machinery that includes several small nuclear ribonucleoproteins (snRNPs).^16,17^ Constitutive splicing of pre-mRNA transcripts produces identical isoforms from the same gene (*Figure 1*) while AS enables different mRNA isoforms to be generated via various mechanisms (*Figure 2*) such as exon skipping, in which one or more exons are skipped and not included in the mRNA transcript.^18,19^ Other common events include mutually exclusive exons, alternative 5´ or 3´ splice sites, and intron retention. Thus, AS of pre-mRNA transcripts can regulate mRNA stability, localization, and translation efficiency.^20–22^ Finally, protein isoforms derived from the host gene by AS can vary in sequence, and consequently differ in structure, localization, or function.^23^ Beyond protein-coding mRNA, the heart’s transcriptome also includes non-coding RNAs (ncRNAs), which can be generated through non-canonical splicing mechanisms.^24^ One prominent class are circular RNAs (circRNAs), which form via a ‘back-splicing’ process that joins a downstream 5′ splice site to an upstream 3′ site, creating a covalently closed loop structure with a back-splice junction unique to the circRNA molecule. CircRNAs are highly stable and often show tissue-, development-, or disease-specific expression.^25,26^ This underpins the principle that RNA splicing in the heart can yield structurally and functionally diverse mRNA or non-coding transcripts from a single gene locus.

### Regulatory networks: splicing factors and non-coding RNAs

AS programs are deeply integrated within broader regulatory networks including epigenetic, transcriptional, post-transcriptional, translational, and post-translational regulation of gene expression. While regulation of splicing factors (SFs) can occur at any of these levels, and subsequently lead to downstream changes in AS, the details of these mechanisms are beyond the scope of this review. This review focuses on regulation of cardiac AS by sequence-specific SFs and non-coding RNAs.

In addition to the core spliceosome machinery, sequence-specific RNA-binding proteins (RBPs) bind to short sequence motifs in pre-mRNA and enhance or repress specific splicing patterns by regulating spliceosome activity or recruitment to splice sites (*Table 2*). Several of these factors display cardiac-specific expression. One of the most well-studied is RBM20, a splicing repressor that suppresses inclusion of specific exons in key sarcomeric (e.g. *TTN*) and calcium-handling genes (e.g. *CaMKIIδ*, *RYR2*, and *CACNA1C*).^27^ Additionally, RBFOX proteins regulate splicing by recognizing a UGCAUG RNA motif, the position of which determines whether splicing is activated or repressed.^35,36,39^ RBFOX1 targets include focal adhesion genes while RBFOX2 targets include sarcomeric and calcium-handling genes. QKI, typically known for its role in the brain, is also highly expressed in developing and adult human hearts and directly targets genes associated with the sarcomeric Z-disc and calcium-handling.^41^ Strikingly, QKI expression is down-regulated in cardiomyocytes under doxorubicin stress and is known to regulate the expression of cardiac circRNAs (*circTtn*, *circFhod3*, and *circStrn3*) in mice, which influence the survival of cardiomyocytes under stress conditions.^42^ Furthermore, CELF proteins, MBNL1, and RBM24 are key regulators of AS during cardiac development.^34,43^ The expression of these factors is tightly regulated throughout development, and their function can be further controlled by post-translational modifications such as phosphorylation, which can alter SF localization, its interaction with other proteins/RNA, and its intrinsic activity. To this end, dysregulation of each of these cardiac-enriched SFs is associated with various forms of CVD.^28^

**Table 2:** List of RNA-Binding proteins regulating alternative splicing events in cardiac development and disease

RNA-binding protein	Key splicing targets	Functional impact	Associated cardiac condition	Ref.
RBM20	*TTN*, *CAMKIIδ*, *RYR2* and *CACNA1C*	Controls sarcomere elasticity and calcium signalling	Dilated cardiomyopathy	Maatz *et al*.^27^; Montañés-Agudo *et al*^28^; Beraldi *et al*^29^; Guo *et al*^30^; Methawasin *et al*^31^; van den Hoogenhof *et al*^32^; Fenix *et al*^33^
RBM24	*ACTN2, FLNC, MYH10*	Orchestrates myofibrillogenesis	Dilated cardiomyopathy	Montañés-Agudo *et al*^28^; Lu *et al*^34^
RBFOX1	Recognize UGCAUG RNA motif in their pre-mRNA targets*, SERCA2, MEF2*	Focal adhesion genes, transcription factor	Pressure overload, myocardial infarction, Cardiac hypertrophy	Sun *et al*^35^; Zhang *et al*^36^; Viereck and Thum^37^; Zorn *et al*^38^
RBFOX2	Recognize UGCAUG RNA motif in their pre-mRNA targets*, SCN5A*, *MEF2D*	Regulates ion channel splicing and transcription factors	Arrhythmias, heart failure	Sun *et al*^35^; Zhang *et al*^36^; Viereck and Thum^37^; Yeo *et al* .^39^; Singh *et al*.^40^
QKI	*Ttn*, *ACTN2, CAMK2D, PDLIM5, NEBL, ABLIM1, RYR2, CACNA1C* and circRNAs, *Morf4l2*	Maintains RNA stability and circRNA biogenesis	Myocardial infarction, remodelling, and cardiac cachexia	Montañés-Agudo *et al* .^9^; Chen *et al*.^41^; Gupta *et al*.^42^
MBNL1	YGCU(U/G)Y motifs in introns, to modulate splice-site selection, *Mbnl1, cTnT, Sorbs1, Ldb3B, Fn1*	Essential for skeletal and cardiac muscle differentiation	Conduction defects, dilated cardiomyopathy	Montañés-Agudo *et al*.^28^; Kalsotra *et al*.^43^
CELF1/2	*TNNT2*, *RYR2, MYH7B*	Developmental splicing transitions	Congenital heart defects	Montañés-Agudo *et al*.^28^; Kalsotra *et al*.^43^

Crucially, ncRNAs form an additional layer of AS regulation in the heart. Long non-coding RNAs (lncRNAs) can act as scaffolds or decoys that influence SF activity or accessibility.^37^ A striking example is the cardiomyocyte-specific lncRNA *TRDN-AS*, transcribed antisense to the Triadin (*TRDN*) gene. *TRDN-AS* associates with serine/arginine-rich (SR) SFs (SRSFs) in the nucleus and is required for their recruitment to *TRDN* pre-mRNA.^44^ Knocking out Trdn-AS in mice causes mis-splicing of *Trdn,* leading to impaired calcium handling and arrhythmias.^44^ Additionally, *TRDN-AS* is elevated in HF, and positively correlated with an increase in the ratio of the cardiac vs skeletal muscle isoform of *TRDN*.^45^ This illustrates how ncRNAs embedded in the genome’s regulatory architecture can maintain proper mRNA splicing of a crucial calcium-handling gene. Other lncRNAs exert similar influence: in the heart, circRNAs derived from the giant *TTN* transcript can bind and modulate splicing regulators. One such circRNA from *TTN*, cTTN1, contains a unique motif that sequesters SF SRSF10 and affects RBM20 localization.^46^ Loss of this circRNA in *in vitro* models disrupts normal splicing of SRSF10 target genes (e.g. *MEF2A* and *CASQ2*) and even *TTN* itself. These findings underscore that the products of non-canonical back-splicing (like circRNAs) can in turn regulate mRNA splicing processes, forming feedback loops within the cardiac transcriptome.

### Developmental splicing programs in cardiomyocytes

**Figure 3:**
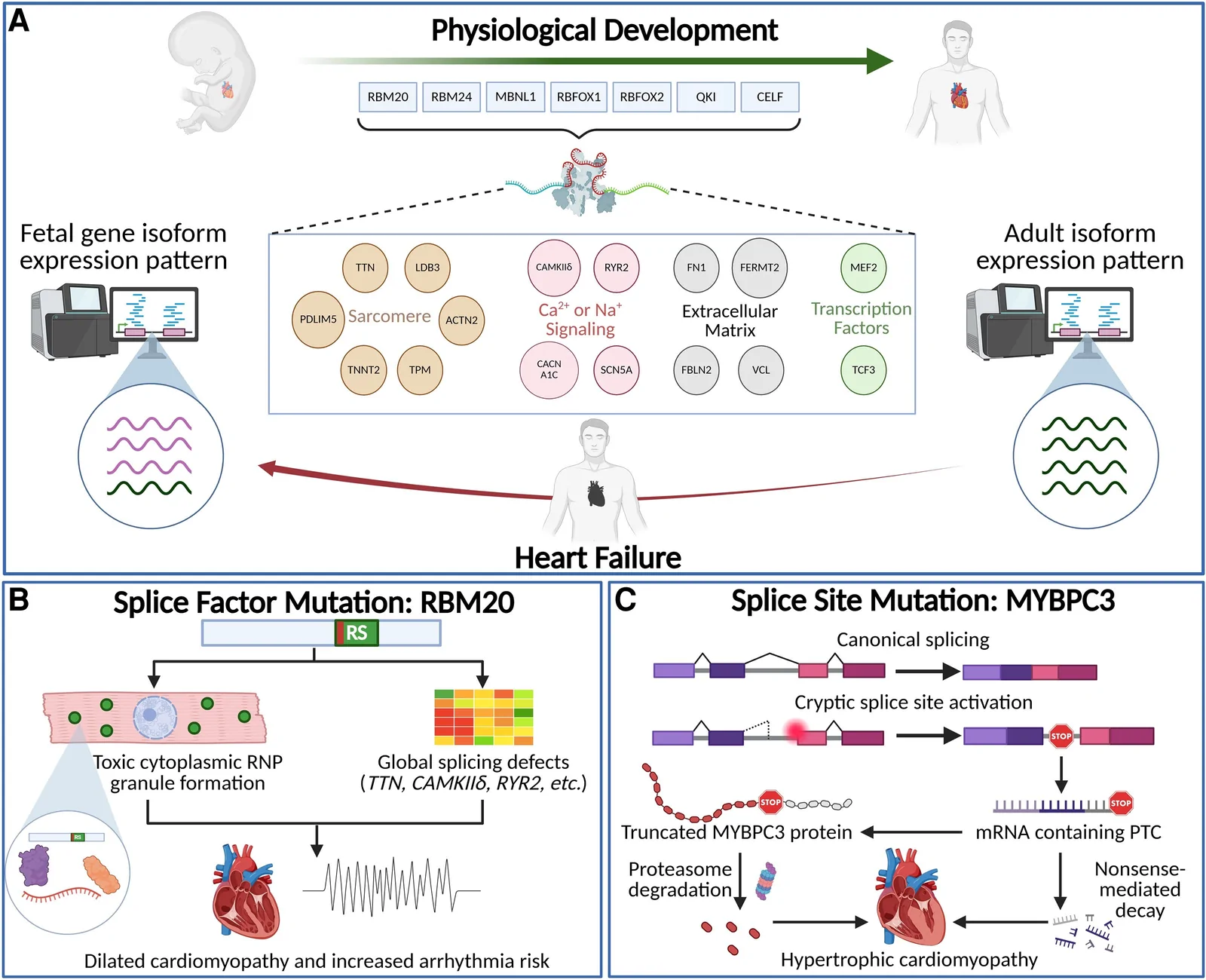
Pathophysiological splicing in cardiovascular diseases. (*A*) Regulators (RBM20, RBM24, MBNL1, RBFOX1/2, QKI, and CELF) of alternative splicing of various cardiac genes orchestrate a switch from neonatal-to-adult isoforms during heart development. Heart failure is often associated with a reversion to foetal isoform expression patterns. (*B*) Mutations within these splicing factors can induce heart failure. For example, patients with RBM20 mutations develop aggressive dilated cardiomyopathy and higher risk of arrhythmia characterized by excessive accumulation of cytoplasmic ribonucleoprotein (RNP) granules and global defects in alternative splicing of mRNA isoforms. (*C*) Mutations affecting splice sites can lead to aberrant alternative splicing of key cardiac genes. For example, mutations in MYBPC3 splice sites can lead to hypertrophic cardiomyopathy due to nonsense-mediated decay of MYBPC3 mRNA containing a premature termination codon (PTC), and/or production of MYBPC3 mRNA containing pre-mature termination codon (PTC) and/or production of aberrant MYBPC3 protein products which undergo proteasome degradation. Created in BioRender. Chatterjee, D. S. (2026) https://BioRender.com/xd10a91

Cardiac remodelling during the foetal-to-adult transition is partially regulated by cardiac-enriched SFs that drive AS transitions in key sarcomeric, contraction, signalling, and transcription factor genes (*Figure 3*).^47^ Titin (TTN), a key determinant of myofibril elasticity and sarcomere structure, experiences increased exon skipping during development. This favours an RBM20-dependent transition from longer, more compliant TTN, to a shorter protein that stiffens the cardiac muscle.^29–31,48,49^ Other sarcomeric genes including *TNNT2*, *TPM1/3*, *ACTN4*, and *MYL6* also undergo developmentally regulated splicing changes to fine-tune sarcomeric function.^50–52^ Genes involved in E-C coupling also undergo AS transitions during development. Splicing of *CACNA1C* leads to dozens of isoforms with different electrophysiological and biochemical properties.^53–55^ Post-natal down-regulation of the CELF family proteins CUGBP1 and CUGBP2 coincides with up-regulation of MBNL1 expression to orchestrate developmental AS transitions. For example, MBNL1 promotes the replacement of *SCN5A* foetal exon 6A with adult exon 6B, modulating development of electrophysiological properties.^56^ In addition, splicing of *CaMKIIδ* is also developmentally regulated, and thought to be controlled by SRSF1, RBFOX1/2, and RBM20.^32,57,58^  *CaMKIIδ* isoforms differ in subcellular localization and calcium-handling properties.^32,57,59^ Finally, a recent study identified hundreds of previously unreported cardiac AS events regulated during the human pre- to post-natal transition.^51^ In addition to genes involved in contractility, these splicing alterations affected extracellular matrix genes, transcription factors (*MEF2D* and *TCF3*), and metabolic genes such as *PKM*. RBFOX1/2 are known splice regulators of MEF2 transcription factors, which are critical for cardiac development and function.^38,40^ Ultimately, this highlights that dynamic temporal regulation of gene expression and function via AS contributes to cardiac maturation.

### Pathological dysregulation of RNA splicing in cardiovascular disease

Pathological splicing patterns can develop from genetic mutations either within an SF, thus disrupting its function, or within a splice site or regulatory element, leading to altered splicing of the associated gene(s). Aberrant splicing can also be triggered by pathological stimuli/stressors that alter SF expression or function. This can manifest either as a change in coding isoform ratios, or the production of isoforms containing pre-mature termination codons, which are degraded by cellular surveillance pathways or lead to dysfunctional truncated proteins. The dysregulated splicing in various CVDs is discussed briefly.

#### Cardiomyopathies (dilated, hypertrophic, and ischaemic)

Gain or loss-of-function mutations in SFs or splice sites, especially in sarcomeric or contraction-associated genes, are associated with various cardiomyopathies (*Figure 3*). RBM20 mutations, predominantly in the RS-domain (arginine and serine-domain), are found in ∼3% of DCM patients,^60^ which lead to RBM20 mis-localization, aberrant splicing of RBM20 targets, and cytoplasmic ribonucleoprotein (RNP) granule formation.^33^ DCM is also associated with splice variants in *TTN*, *LMNA*, and *DSP.* The most common genetic cause of DCM is *TTN* truncation variants (*TTN*tv), which often stem from splice-site mutations. *TTN*tv are either degraded or incorporated into the sarcomere, which leads to increased cardiomyocyte stiffness and eventually DCM.^61^ In hypertrophic cardiomyopathy (HCM), mutations in *MYBPC3* are the most prominent pathogenic variants, with a large portion affecting splice sites.^62,63^  *MYBPC3* variants lead to cryptic splice site activation, exon skipping, and/or pre-mature termination codons which can lead to non-sense-mediated mRNA decay (*Figure 3*). Furthermore, ischaemic cardiomyopathy (ICM) and DCM are associated with an increase in TTN isoform size, a foetal-like reversion that corresponds with decreased myocardial stiffness.^64,65^ Although, ICM derives from myocardial infarction (MI) or coronary artery disease, a recent study of DCM and ICM cohorts observed largely indistinguishable transcriptomes between the two.^66^ This suggests that end-stage HF converges on a common isoform landscape, regardless of the underlying disease aetiology.^66^ CircRNA expression is also dysregulated in cardiomyopathy.^67,68^ For example, *circSLC8A1* is abnormally up-regulated in DCM patients and has been demonstrated as a potential therapeutic target.^69^ In mice pathological accumulation and nuclear retention of *circDhx32* was observed after ischaemia reperfusion injury (IRI).^70^

#### Arrhythmogenic disorders

Arrhythmogenic disorders can also result from splicing defects in cardiac ion channels, such as *SCN5A*, which encodes the cardiac sodium channel. HF is associated with aberrant *SCN5A* mRNA splicing that produces truncated channel isoforms, and higher levels of these pathological splice variants correlate with electrical remodelling and increased ventricular arrhythmias.^71^ Splice-altering variants in *SCN5A* and the potassium channels *KCNQ1* and *KCNH2* have been identified in patients with Brugada syndrome and Long QT syndrome.^72^ Because RBM20 mutations disrupt splicing of calcium-handling genes such as *CaMKIIδ* and the cardiac ryanodine receptor *RYR2*, these patients also display increased risk of arrhythmias.^32^

#### Atherosclerosis

AS dysregulation has also been linked with the pathogenesis of atherosclerosis, a chronic inflammatory disease underlying cardiovascular events. In a cohort of patients that underwent carotid endarterectomy, decreased expression of the *FOXP3Δ2* isoform was associated with atherosclerotic plaque instability.^73^ Mechanistic studies demonstrated that Treg cell activation led to increased FOXP3Δ2 expression and subsequent activation of a protective transcriptional program that may combat atherosclerosis. Another study observed that increased *STAT3β* isoform expression ultimately promoted a macrophage-like phenotype in vascular smooth muscle cells (VSCMs), which contributes to atherosclerotic plaque vulnerability.^74^ This effect was driven by decreased expression of the SF PCBP1. In addition, transcriptome-wide analysis identified >5000 AS events between early and advanced stage atherosclerosis fibroatheromas, providing a valuable dataset for understanding the link between atherosclerosis progression and AS.^75^ CircRNAs also play a role in atherosclerosis. *CircANRIL* originates from the chromosome 9p21 locus, which is one of the strongest genetic risk regions for atherosclerosis. *CircANRIL* negatively regulates ribosome maturation and induces apoptosis in VSCMs and macrophages which ultimately prevents atherosclerosis.^76^ Another study identified *circLONP2*, as a shear stress-sensitive, pro-atherogenic regulator, which induces oxidative stress and endothelial inflammation, thereby accelerating atherosclerosis.^77^ RNA profiling of small extracellular vesicles (EVs) released by monocytes from patients with coronary atherosclerotic disease, identified *CircNPHP4*, as a key regulator of vascular inflammation and plaque development.^78^

#### Congenital heart disease

As discussed above, many RBPs critically regulate AS to drive development and pathogenic mutations affecting these factors can lead to congenital heart disease (CHD). For example, multiple large-scale studies of CHD cohorts have identified an association between *RBFOX2* mutations and CHD, such as hypoplastic left heart syndrome.^79,80^  *RBM10* mutations have been linked with TARP Syndrome, a multifaceted developmental disorder which is partly characterized by congenital heart defects.^81^ Furthermore, splice-altering variants affecting sarcomeric genes, transcriptional regulators (*TBX5*, *GATA4*, and *ZIC3)*, and conduction genes (*KCNH2*) have been associated with multiple CHDs, including Marfan’s syndrome, heterotaxy, septal defects, and congenital Long QT syndrome.^81,82^ Identifying CHD-associated variants and the molecular mechanisms linking these variants with CHD will allow for more informed pre-natal genetic screening, and potentially novel therapeutic approaches.

#### Heart failure

HF is the chronic end-stage of various CVDs, characterized by impaired pumping and a reversion to foetal-like metabolic and oxygen consumption states. This is associated with reactivation of foetal-specific RNA-binding proteins (QKI, RBFOX2, RBM24, etc.) and thousands of foetal-specific mRNA isoforms.^83^ Similar reactivation of foetal splicing program has been observed in models of pathological cardiac hypertrophy.^13^ This evidence underscores the role of AS in the onset and progression of cardiomyopathies and HF.

## Identification of splicing signatures and their application as biomarkers

### Bulk and single-cell transcriptomics

Short-read (50–300 bp) RNA-sequencing (RNA-Seq), a high-throughput and now relatively inexpensive technology has enabled systematic profiling of AS in cardiac development and disease.^84–90^ However, it is difficult to reconstruct full transcripts using short-read RNA-Seq, especially for those that are longer or undergo more complex AS. Long-read sequencing technologies (Oxford nanopore technology (ONT) and Pacific Biosciences (PacBio)) have solved this problem by facilitating sequencing of full transcripts in a single read.^91–93^ However, higher cost, lower read output, and increased error rate when compared with short-read RNA-Seq limit its applications.^91^ Single-cell RNA-sequencing (scRNA-Seq) can dissect cell-type specific regulation of gene expression and AS in cardiac development and disease.^94^ Unlike bulk RNA-Seq, scRNA-Seq can capture cellular heterogeneity due to differentiation state/cell type or differing responses to stress or therapeutic intervention. Technical challenges exist, as cardiomyocytes are too large for standard high-throughput droplet-based single-cell methods. Alternatives that overcome this problem include SMART-Seq, SPLiT-Seq, or sequencing from isolated single nuclei as a proxy for whole cells.^95,96^ Recently, a comprehensive splicing atlas of the adult human left ventricle was constructed by combining single nuclei isolation with ONT sequencing of healthy and ischaemic HF patient samples.^97^ This work identified cell type-specific differences in isoform expression and disease-associated isoform switching linked to cardiomyocyte and fibroblast remodelling that was invisible by bulk RNA-Seq analysis. Continued development of scRNA-Seq technologies will help overcome current technical barriers and enable precise characterization of cell-specific isoform expression patterns in various CVD types and stages.

### Mendelian randomization

Mendelian randomization (MR) is an epidemiological method used to assess causal relationships between risk factors and CVD^98^ by using genetic variants as proxies for risk factor exposure and comparing outcomes of cohorts of individuals with different genetic variants. For example, identifying a genetic variant associated with higher LDL cholesterol and with increased CVD risk would support the notion that LDL causally increases CVD risk. The three major assumptions of MR are that the genetic variant is (i) associated with the exposure, (ii) not associated with confounders, and (iii) affects the outcome only through the exposure. Despite the limitations associated with these assumptions, MR allows for greater causal inference and bias elimination compared to observational studies. Thus, when randomized clinical trials are infeasible, unethical, or costly, MR offers an improved approach for identifying CVD risk factors. While most studies have leveraged gene expression quantitative trait loci (QTL), a recent study also incorporated splicing QTL data to identify key splicing loci associated with coronary heart disease risk.^99^ Future studies using similar approaches will further define links between AS, risk factors, and CVD.

### Integration of multi-omics and clinical data

AS does not act in isolation and integrating splicing data with other molecular and clinical information can yield a more comprehensive understanding of the origin and progression of CVD.^100^ A recent integrative analysis of human heart tissues used RNA-Seq to guide mass spectrometry, uncovering over 200 previously unannotated protein isoforms generated by AS.^101^ This highlights that measuring the proteomic consequences of mRNA splicing may aid in assessing the functional impact of isoform switches. Furthermore, incorporating gene expression and AS analysis of human HF RNA-Seq data with machine-learning approaches identified over 4000 abnormal splicing events and revealed several splicing regulators (e.g. RBM5, ZRANB2, and hnRNP F/A0) as central nodes in HF.^102^ Transcriptome-wide association studies (TWAS) integrate genetic variant and gene expression information to pinpoint genes associated with disease phenotypes.^103,104^ Splicing-based TWAS offers major benefits by capturing genetic effects on an AS level that traditional expression-based TWAS approaches often miss,^105^ thus identifying new susceptibility genes and AS events for CVDs.

Incorporating RNA-Seq and genomic sequencing data has led to the development of deep-learning tools to improve the prediction and detection of pathogenic splice-altering variants (*Table 3*). These tools can better identify intronic, non-coding, or cryptic mutations that disrupt splicing. In this context, researchers have developed heart-specific splicing prediction models to analyse patient genomes, enhancing the yield of genetic diagnosis for CVDs.^107^ Additionally, integrating RNA-Seq from SF knockout studies and crosslinked-immunoprecipitation data to identify SF-binding motifs has led to the development of models designed to predict SF regulation and targets.^106^ Such models can even infer relevant SFs from clinical RNA-Seq data. As more clinical and research-generated datasets become available for training these models, the power and accuracy of such machine-learning tools will continue to grow.

**Table 3:** List of useful tools for interpreting patient sequencing data in the context of cardiac alternative splicing

Tool	Description	Website [reference]
SpliceAI	Deep-learning algorithm for predicting the effect of genetic variants on splicing.	https://github.com/Illumina/SpliceAI ^86^
SpliceAI Lookup	User-friendly website for obtaining SpliceAI, Pangolin, and additional predictor scores for genetic variants of interest.	https://spliceailookup.broadinstitute.org/ ^88^
Pangolin	Deep-learning algorithm for predicting the effect of genetic variants on splicing. Trained on tissue-specific data.	https://github.com/tkzeng/Pangolin ^87^
Heart isoform atlas	Online graphical user interface webserver containing snRNA-Seq data from healthy and heart failure patient cohorts. Can be used to assess isoform composition between cell types in healthy and diseased states.	https://github.com/gaolabtools/heart-isoform-atlas ^97^
Cardiodb.org	Provides a collection of tools for analysing genetic variants in cardiovascular disease.	https://www.cardiodb.org/ ^89^
ClinVar	Public archive of genetic variants and their association with disease.	https://www.ncbi.nlm.nih.gov/clinvar/ ^90^
KATMAP	Computational tool for modelling how splice factors regulate splicing by integrating RNA-seq data with splice factor binding motifs. Can predict direct splicing factor targets, identify position-specific regulatory effects, and infer relevant splice factors from clinical sequencing data.	https://gitlab.com/LaptopBiologist/katmap ^106^

### AS-related circulating biomarkers

While many studies have characterized pathological splicing changes in the heart, it is inherently challenging to obtain cardiac biopsies from patients to detect altered isoform expression. Thus, RNA extraction from peripheral blood samples is a feasible alternative. Altered levels of circRNAs derived from multiple genes (*FNDC3b*, *NCX1*, and *HIPK3*) have been identified in blood samples of patients with various CVDs.^108^ Another circRNA, *MICRA*, was identified to be highly predictive of left ventricular dysfunction after MI.^109^ Analysis of RNA extracted from blood of HCM patients harbouring an *MYBPC3* mutation identified increased skipping of *MYBPC3* exon 7, corroborating observations from heart biopsies.^63^ In another pilot study of HF patients, circulating levels of two truncated SCN5A splice variants were strongly associated with appropriate implantable cardioverter-defibrillator shocks, enabling excellent discrimination of patients at risk.^110^ Importantly, the abundance of these circulating splice variants mirrored their levels in myocardial tissue and decreased in patients who responded to therapy, underscoring their potential as minimally invasive biomarkers of disease severity.^110^ Future studies should continue to identify and validate circulating RNA biomarkers in different disease contexts, followed by incorporation of such data into routine diagnostic or risk stratification algorithms.

### Prognostic and diagnostic applications

**Figure 4:**
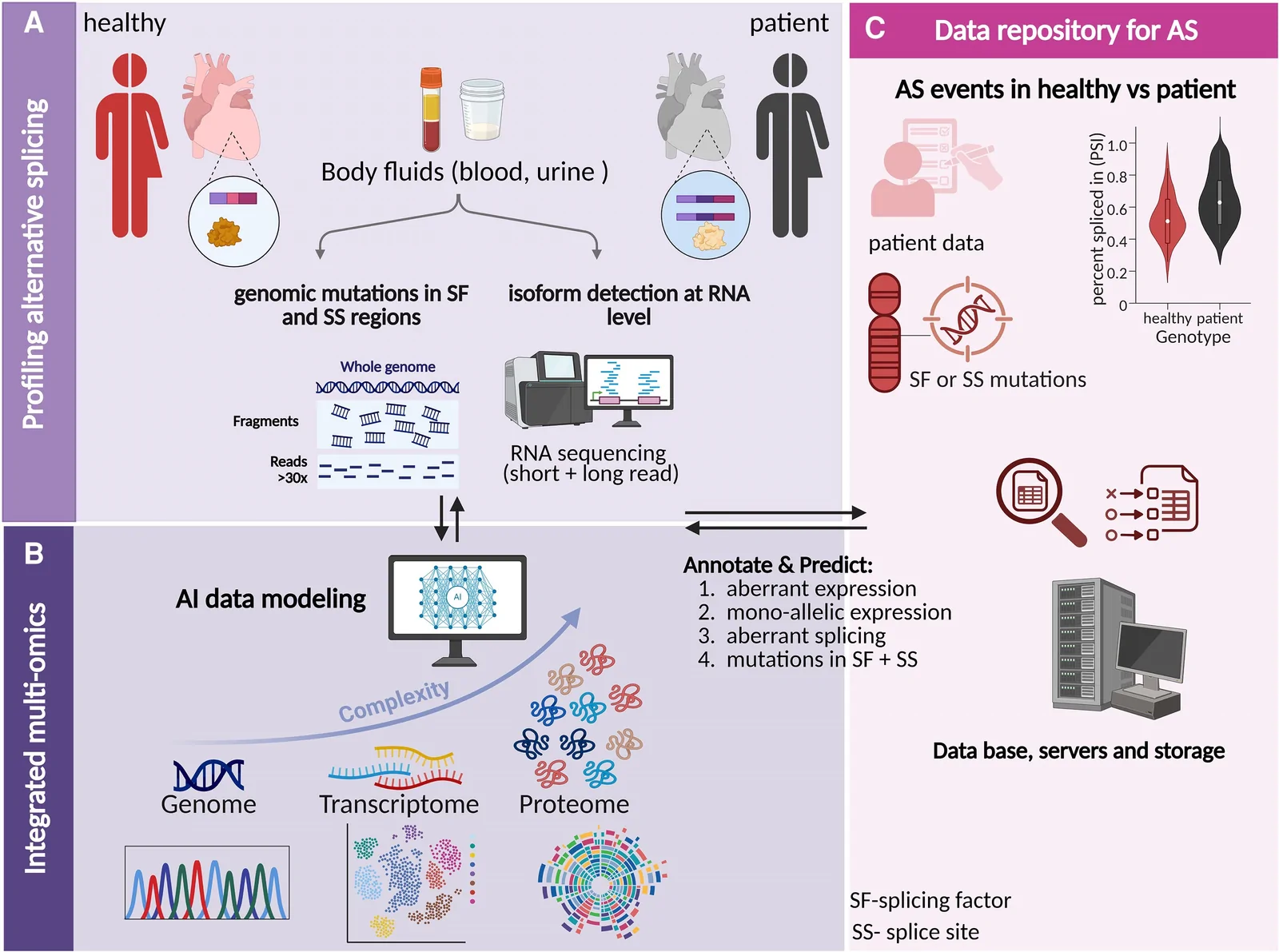
Diagnostic potential of splicing signatures in cardiovascular diseases. (*A*) Genetic information encoded in precursor RNA can be transcribed into coding sequences, non-coding regulatory elements, or undergo alternative splicing events that diversify transcript and protein outcomes between healthy volunteers and heart failure patients. Detection of circulating RNA isoforms by non-invasive collection of body fluids (blood, urine) from heart failure patients, can expedite clinical decision-making. (*B*) Functional validation of suspected splice factors (SFs), splice sites (SSs), or regulatory mutations can be performed in cell culture (splicing reporter assays or gain-of-function or loss-of-function assays). Integration of multidimensional molecular data collected at the genomic, transcriptomic, and proteomic level from well-characterized patient cohorts or cell culture experiments is crucial. (*C*) Alternative splice factors or coding and non-coding RNA isoforms identified and characterized from profiling and multi-omics are essential to build data repositories which can be used for annotation and prediction. In silico (computational) methods, such as data mining, machine learning, and deep learning, can also be used to aid diagnosis and multiomic analysis. Artificial intelligence (AI), machine learning algorithms could be developed and validated that use both transcript information and genome variation to predict cryptic splice sites and possible effects on protein structure. Created in BioRender. Chatterjee, D. S. (2026) https://BioRender.com/ingjkb8

Emerging evidence indicates that specific RNA splicing signatures can aid in distinguishing diseased hearts and even predict disease progression in patients. Global splicing analyses in failing human myocardium have revealed widespread AS changes, particularly in sarcomere genes (e.g. *TNNT2*, *TNNI3*, *MYH7*, and *FLNC*), in both ICM and DCM.^111^ Notably, in the context of aortic stenosis, some of these splice alterations were detectable even before the onset of overt HF, highlighting their potential as indicators of early onset of the disease.^111^ The relative expression of foetal vs adult isoforms can serve as a ‘molecular fingerprint’ of pathology. For example, isoform ratios of TNNT2 and MYH7 were shown to classify diseased vs healthy hearts with >98% accuracy in a blinded analysis.^111^ A recent study reported that elevated expression and phosphorylation of the nuclear *CAMKIIδ-B* splice isoform were inversely correlated with a functional recovery response to left ventricular assist device therapy.^112^ Such findings suggest that panels of splicing events could function as highly specific diagnostic biomarkers for cardiomyopathies (*Figure 4*).

## Therapeutic modulation of cardiac RNA splicing

Treatment for disease causing AS events is challenging due to the complicated molecular pathology. Patient management ranges from treating symptoms (e.g. HF and arrhythmias) to implanting assist devices or, as a last resort, heart transplantation. The following section emphasizes the promising therapeutic approaches and the barriers to their implementation for treating AS-derived CVD in patients.

### Antisense oligonucleotides

**Figure 5:**
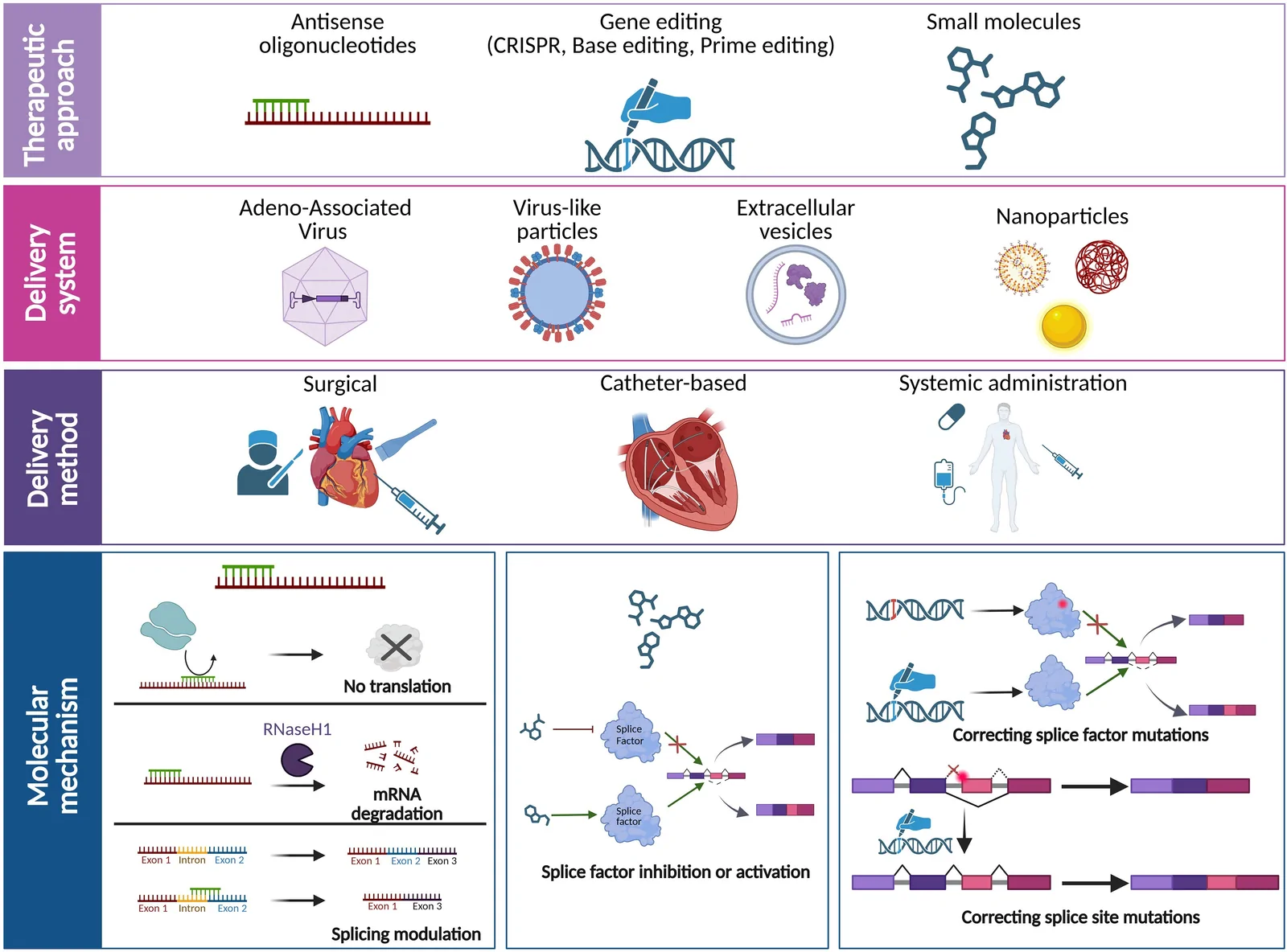
Therapeutic strategies targeting RNA splicing for treatment of heart failure. Therapeutic methods available to modulate splicing processes include the use of anti-sense oligonucleotides, gene-editing tools such as CRISPR or small molecules. Optimization of delivery systems (viral or non-viral) and administration routes (surgical vs systemic application) should be considered to achieve effective targeting of dysregulated alternative splicing events for the treatment of heart failure. Anti-sense oligonucleotides can prevent translation or promote degradation of pathological isoforms, as well as directly modulating splicing by binding to splice junctions. Small molecules can be used to inhibit or activate splicing factors which in turn lead to modulated splicing of transcripts regulated by these factors. Gene editing approaches can be used to correct mutations in splice factors and restore proper function or correct splice-site mutations that cause dysregulated splicing of key cardiac genes. Created in BioRender. Chatterjee, D. S. (2026) https://BioRender.com/7i3z78r

Antisense oligonucleotides (ASOs) are short synthetic oligonucleotides designed to bind specific RNA sequences and regulate gene expression via steric inhibition, promoting RNA degradation, or modulating splicing (*Figure 5*). Although no ASO therapy currently exists that treats CVD by modulating AS, several studies have demonstrated the potential of this approach. In a mouse model of HF with preserved ejection fraction (HFpEF), treatment with RBM20–ASOs increased the expression of longer, more compliant TTN isoforms, improved diastolic function, and normalized ventricular dimensions.^113^ In another study, treatment of patient-derived human induced pluripotent stem cell-derived cardiomyocytes (hiPSC-CMs) with ASOs inhibited HCM-causing *MYBPC3* cryptic splicing and restored canonical splicing.^114^ Another study delivered ASOs into a *Mybpc3*-targeted knock-in mouse model of HCM.^115^ These ASOs masked splicing enhancer motifs within exons 5 and 6 and successfully reduced aberrant mRNA expression while abolishing systolic dysfunction and inhibiting cardiac hypertrophy. These studies validate the concept of modulating AS with ASOs to treat cardiac disease.

### Small molecules

Few studies have investigated the usage of small molecules as therapeutic modulators of AS in the heart. Cardenolides are a class of cardiotonic steroids that inhibit Na^+^/K^+^-ATPase and have been used in the treatment of HF and atrial fibrillation.^107^ A screen of ∼34 000 small molecules identified several cardenolides that altered expression of splicing related genes, reduced RBM20 expression, and ultimately inhibited RBM20-mediated *TTN* splicing.^116^ While reducing RBM20 improves diastolic function in mouse models of HFpEF, cardenolide doses required to achieve significant depletion resulted in toxicity *in vivo*.^31,116,117^ Treating cells with *all-trans* retinoic acid up-regulated RBM20 and subsequently alleviated DCM phenotypes in hiPSC-CMs.^118^ As our understanding of cardiac regulatory splice networks continues to grow, translational research is needed to identify small molecules that can modulate SF expression/localization/function or directly target splice sites themselves.

### Genome editing

Genome editing to correct underlying pathogenic mutations represents a promising, yet technically challenging approach to potentially curing genetic CVD. Technologies such as CRISPR genome editing, base editing, and prime editing can be used to correct pathogenic genomic mutations and have shown promise in model systems of CVD.^119^ Chemello *et al*. used base and prime editing to modify splice acceptor/donor sites in hiPSC-CM and a murine model of Duchenne muscular dystrophy (DMD) containing an exon 51 deletion in the dystrophin gene.^120^ This mutation introduces a pre-mature termination codon in exon 52, leading to a non-functional dystrophin isoform and the development of DMD in patients. By promoting skipping of exon 50 or 52, or reframing exon 52, they were able to restore functional dystrophin expression in cardiomyocytes. More recently, base editing was used in hiPSC-CMs and mice to correct pathogenic DCM-causing RBM20 RS-domain mutations.^121^ The mutant phenotype of mis-localized cytoplasmic RBM20, toxic RNP granule formation, and dysregulated AS was rescued after base correction. However, to avoid off-target genome editing, future work is required to enhance the precision and safety of these tools before they can be clinically translated.

### Delivery challenges

Identifying strategies that allow for robust and tissue-specific delivery while avoiding negative consequences stemming from the delivery vectors themselves remain a central challenge for cardiac therapeutics. Several main delivery packaging strategies exist: recombinant adeno-associated virus (AAV), nanoparticles, virus-like particles (VLP), and EVs^119,122^ (*Figure 5*). AAVs are small non-pathogenic viruses that can be modified to carry therapeutic cargo. AAV-mediated delivery of base editing machinery or ASOs has been used to treat mammalian models of HCM, DCM, MI, dystrophic cardiomyopathy, and Barth syndrome.^115,123–128^ Nanoparticles are a non-viral alternative that have been used to deliver gene-editing machinery into various tissues in mice and monkeys.^119,129–132^ Recently, VLPs have emerged as a novel delivery vehicle. Unlike AAVs, VLPs consist only of the viral scaffolds that package cargo proteins, RNPs, or mRNAs.^133^EVs, including exosomes and microvesicles, are also being explored as natural nanocarriers for delivery of therapeutic material.^134^ In addition to packaging systems, multiple delivery methods exist which can be divided into three categories: surgical, catheter-based, and systemic administration. These approaches and their advantages/disadvantages have been reviewed elsewhere.^122^

Significant challenges remain for efficient, safe, cardiac-specific delivery of therapeutics. Surgical and catheter-based delivery methods directly deliver therapeutic agents to the heart but are invasive or technically challenging,^122^ while systemic administration is more likely to have off-target effects in other organs. Modifying delivery vectors to make them more cardiotropic is a promising approach. AAV transduction of non-target organs is undesirable and potentially toxic, as evidenced by the development of subacute liver failure in spinal muscular atrophy (SMA) patients treated with an AAV-based therapy.^135^ Recently, several studies have engineered novel AAV-derived vectors that demonstrate decreased liver transduction while maintaining efficient cardiac muscle targeting.^136,137^ Cardiac-specific promoters have been used to ensure tissue-specific expression, and protease-activatable AAV vectors have been used to specifically target damaged regions of the heart.^119,138^ Delivery of nanoparticles and EVs is typically less efficient than AAVs and results in high uptake by other tissues, necessitating local delivery or surface engineering efforts to improve cardiac targeting after systemic administration.^130^ Recent work has led to engineered VLPs with improved cargo packaging and delivery and minimal off-target effects.^119,133,139,140^

Another challenge is overcoming immune responses that mitigate delivery efficiency, such as pre-existing circulating neutralizing antibodies targeting AAV capsids. Direct injection of AAVs into the heart, pre-emptive depletion of these antibodies, and encapsulating AAVs in exosomes are strategies to address this problem.^141–145^ Modifying the AAV genome by removing specific sequences or incorporating immune-inhibitory sequences has also been shown to promote immune system evasion, and may improve the efficacy of AAV-based therapies.^141,146–148^ Nanoparticles typically have lower immunogenicity than AAVs, and can be engineered to further lower their immunogenicity and facilitate the delivery of different types of cargo (mRNA^119^ and circRNA^149^). Consequently, nanoparticles are currently more suited for repeat administration of therapeutics compared with AAVs. Similarly, EVs and VLPs can be engineered to escape immune responses.

While promising strides have been made, significant work remains to be done to develop safe, effective, and ideally non-invasive delivery methods for cardiac-targeting therapeutics. Bio-engineering efforts should continue to focus on improving delivery vector cardiotropism and reducing immunogenicity. Furthermore, *in vivo* studies in mammalian models are critical before clinical trials can proceed.

### Lessons from neuromuscular disease therapies

While no such therapy has been approved or is in clinical trials, the most compelling evidence that splicing modulation can be translated clinically for the treatment of CVDs comes from neuromuscular disease therapies. Nusinersen is an ASO-based medication administered by intrathecal injection that is used to treat SMA by enhancing exon 7 inclusion in the *SMN2* gene, restoring levels of functional SMN protein.^150^ Risdiplam is a small-molecule oral medication that operates by a similar mechanism targeting exon 7 and received FDA approval in 2020.^151^ Eteplirsen and golodirsen are exon-skipping ASO medications that restore the reading frame of the dystrophin transcript in patients with DMD-causing dystrophin mutations.^152^ The success of these therapies suggests that splicing modulation can be both safe and efficacious in patients, establishing a basis for treating CVDs via similar mechanisms.

## Conclusion and future perspectives: translating AS-based research into clinics

RNA splicing has emerged as a critical layer of gene regulation governing key aspects of cardiac development, homeostasis, and disease progression. The integration of multi-omics technologies has unveiled complex splicing networks regulated by RNA-binding proteins and ncRNAs, and highlighted their importance in HF. These insights are now being translated into actionable clinical tools, including splicing-based diagnostics and circulating biomarkers that may offer early detection and improved prognosis of CVDs. However, there is a significant need for the development of easily accessible databases linking CVD phenotypes with biomarker levels and standardized, rapid biomarker tests that are quicker and cheaper than RNA-Seq, which will enable more accessible healthcare and reduce financial burdens. Similarly, while splice prediction tools have improved greatly, it is critical that easily accessible portals such as SpliceAI Lookup^88^ are maintained to make utilizing these tools more accessible for clinicians.

While sequencing and splice variant prediction efforts can uncover signatures of CVD, identifying therapeutic targets requires in-depth analysis to validate pathogenicity and establish therapeutic feasibility. Validation in hiPSC-CMs is one approach to study the functional effects of splicing mutations in cardiomyocytes. Gene-editing existing iPSC lines or obtaining cells directly from patients with CVD-associated mutations, the functional effects of genomic mutations can be verified. However, in addition to being time-consuming and costly, monolayer hiPSC-CM cultures do not fully recapitulate the adult human heart, highlighting the need for more complex *in vitro* platforms such as human cardiac organoids or engineered heart tissues.^51^

Therapeutic approaches that target aberrant splicing have entered pre-clinical validation and may provide future individualized treatments for patients with genetically or functionally defined splicing defects once approved for safety by the regulatory authorities. The successful translation of splicing-targeted therapies for muscular dystrophy and SMA provides a strong rationale to pursue similar strategies for heart disease. Validating therapeutic approaches in hiPSC-CMs or animal models is critical to identify any off-target effects of the therapeutic itself, such as CRISPR editing of non-target genomic regions. Furthermore, the research community should focus on bio-engineering efforts to develop effective, specific, and non-immunogenic cardiac therapeutic delivery systems. Although therapeutic interventions targeting AS to treat CVD are still in early stages of development and must overcome major safety challenges, they hold a great deal of promise.

## Declarations

### Data availability

No data were generated or analysed for or in support of this paper.
